# Perioperative Seizures and Quality of Life in Falx and Convexity Meningiomas: Key Factors of Patient Outcomes

**DOI:** 10.3390/cancers17071174

**Published:** 2025-03-31

**Authors:** Alim Emre Basaran, Martin Vychopen, Erdem Güresir, Johannes Wach

**Affiliations:** 1 Department of Neurosurgery, University Hospital Leipzig, 04103 Leipzig, Germanyerdem.gueresir@medizin.uni-leipzig.de (E.G.);; 2 Comprehensive Cancer Center Central Germany, Partner Site Leipzig, 04103 Leipzig, Germany

**Keywords:** meningioma, health-related quality of life, epilepsy, peritumoral edema

## Abstract

Background: Falx and convexity meningiomas can compress surrounding brain structures, frequently resulting in epileptic seizures, which adversely impact the quality of life (QoL) of affected patients. This study aimed to assess postoperative QoL in patients with documented perioperative seizures associated with falx and convexity meningiomas. Methods: The Quality of Life in Epilepsy Inventory-31 (QOLIE-31) was administered to patients who underwent surgery for falx/convexity meningiomas and experienced perioperative seizures. Seizures were defined as those occurring within 30 days pre- or post-surgery. Results: A total of 77 patients responded to the questionnaire, of whom 44 were female and 33 were male. Multivariable analysis showed that falx meningioma was associated with a lower QOLIE-31 score (OR = 4.02, 95% CI: 1.14–14.18, *p* = 0.03). Furthermore, the presence of peritumoral edema was also associated with lower QOLIE-31 scores (OR = 3.89, 95% CI: 1.04–14.47, *p* = 0.043). Conclusions: This study demonstrates that perioperative seizures in patients with falx meningiomas significantly impact postoperative quality of life. The presence of peritumoral edema was also associated with poorer QoL outcomes. These findings underscore the importance of targeted management strategies to to improve QoL in meningioma patients experiencing seizures.

## Simple Summary

This study examines the impact of perioperative seizures on the quality of life (QoL) in patients with falx and convexity meningiomas. Seventy-seven patients were surveyed using the QOLIE-31 questionnaire after surgery. The results reveal that patients with falx meningiomas and peritumoral edema have significantly lower QoL scores. Additionally, older age at surgery and lower preoperative Karnofsky Performance Scores correlate with reduced QoL. In the multivariate analysis, the falx tumor location and the presence of edema emerged as independent predictors of poorer postoperative QoL. These findings underscore the need for tailored treatment strategies to minimize seizure-related complications and enhance long-term outcomes for meningioma patients.

## 1. Introduction

Meningiomas are among the most common types of intracranial brain tumors [1]. The most preferred locations for meningiomas are the convexity and falx. Together, these locations account for approximately 32% of all meningiomas [2]. Convexity and falcine meningiomas, located on the brain’s outer surface and along the falx cerebri, are classified as non-skull base meningiomas [3]. These non-skull base meningiomas, particularly those situated at convexity and falx regions, are often associated with higher World Health Organization (WHO) grades than their skull base counterparts [4,5,6]. These non-skull base meningiomas are also associated with higher rates of seizures compared to skull base meningiomas. In their systematic review, Englot et al. showed that non-skull base meningiomas were significantly more likely to cause seizures than skull-base meningiomas. Additionally, the same study demonstrated that parasagittal and convexity meningiomas had the highest rates of preoperative seizures [7]. The primary treatment for meningiomas is surgical resection, typically guided by the Simpson classification system, which outlines the extent of tumor removal and correlates with recurrence rates [8].

Although many meningioma patients report an improved health-related quality of life (HR-QoL) after surgery, numerous patients face significant long-term challenges that continue to impact their lives [9]. Among these challenges, seizures are a frequent clinical manifestation in meningioma patients, presenting both before and after surgery [10,11]. The occurrence of seizures can profoundly affect the QoL of meningioma patients. Studies have shown a strong association between seizure activity and limitations in daily activities, underscoring the impact on patient’s functional independence and overall well-being [12,13,14].

Previous studies investigating the relationship between epilepsy and QoL in meningioma patients have primarily used general HRQoL questionnaires, such as the SF-36, EQ-5D, and FACT-Br [15,16]. While these tools offer valuable insights into patients’ general well-being, they lack the specificity needed to capture epilepsy-related aspects of QoL. This limitation suggests the need for focused tools like the Quality of Life in Epilepsy-31 (QOLIE-31) questionnaire, which is sensitive to epilepsy’s unique impact on patients [17,18,19].

To our knowledge, no previous study has specifically examined HRQoL in patients with falx and convexity meningiomas using specialized tools such as QOLIE-31. Previous studies examining the impact of seizures on QoL have typically included heterogeneous groups of meningioma patients without specific emphasis on the distinct subgroups according to tumor location. However, given the unique clinical and surgical challenges associated with falx and convexity meningiomas—including their higher likelihood of seizure occurrence and complex perioperative management—it is essential to evaluate these subgroups individually [3,7]. Our study therefore specifically addresses this knowledge gap by using the QOLIE-31 questionnaire to assess the epilepsy-related QoL impacts in this particular subgroup of meningioma patients. The present study, therefore, aims to evaluate the impact of seizures on QoL in meningioma patients and to identify the key factors that influence postoperative outcomes.

## 2. Materials and Methods

This retrospective study screened patients who underwent surgical treatment for convexity, parasagittal, or falx meningiomas at the Department of Neurosurgery, Medical University of Leipzig, between 2016 and 2021. Convexity meningiomas are defined as tumors arising from the dura mater and overlying the cerebral convexities [20]. Parasagittal meningiomas were defined as tumors located in the parasagittal angle that extends to and/or invades the superior sagittal sinus (SSS) [21]. Falx meningiomas were defined by Cushing as meningiomas arising from the falx cerebri and completely concealed by the overlying cortex [22]. Patients were identified through a retrospective review of institutional medical records, including clinical documentation, radiological imaging, and histopathological reports.

### 2.1. Inclusion and Exclusion Criteria

All patients who met the inclusion criteria and had documented perioperative seizures, which were defined as seizures occurring within 30 days before or after surgery based on neuropathological and neuroradiological examinations and agreed to participate, were included. Neuropathological classification followed the 2016 World Health Organization (WHO) classification of tumors of the central nervous system [23]. To ensure consistency and minimize selection bias, patient records were systematically screened using predefined inclusion criteria. Patients without perioperative seizures were not included in this analysis, as our objective was to specifically assess seizure-related QoL outcomes. Inclusion criteria required patients to be over 18 years of age, have no additional intracranial pathologies, have no history of prior cranial radio and chemotherapy, availability of preoperative and postoperative MRI scans, and completion of the QOLIE-31 questionnaire for postoperative QoL assessment. Additionally, patients were required to have documented perioperative seizures within 30 days before or after surgery, confirmed via medical records or postoperative follow-up. All patients underwent a tumor conference following surgery, where treatment decisions were made based on confirmed neuropathological examination. The conclusion regarding post-surgical treatment or no treatment was reached collaboratively with radiation oncologists, neuro-oncologists, and neuroradiologists. None received postoperative radiotherapy or chemotherapy at the time of data collection due to individual clinical decisions, lack of radiographic progression, or patient preference. These cases were closely monitored with follow-up imaging. No additional adjuvant therapies were recorded in the cohort, ensuring a homogenous sample patient selection was conducted by independent review by two neurosurgeons and a neuroradiologist to minimize selection bias.

### 2.2. MRI Evaluation

All patients underwent preoperative and postoperative MRI scans performed on a 3.0 Tesla scanner using standardized institutional imaging protocols. All imaging analyses were conducted using preoperative and immediate postoperative MRI scans. Imaging from the most recent follow-up was not included in the statistical analysis to ensure consistency and avoid confounding factors related to tumor recurrence or delayed postoperative changes. The tumor location, volume, surface area, and peritumoral region were assessed using preoperative magnetic resonance imaging (MRI). MRI sequences were analyzed with 3D Slicer software (version 5.2.1, Surgical Planning Laboratory, Harvard University, USA), utilizing gadolinium-enhanced T1 sequences for determining tumor location, tumor volume, and surface area. Peritumoral vasogenic edema was assessed in hyperintense areas on T2/FLAIR-weighted MR images. Segmentation methods were performed as previously described by Wach et al. [24]. Preoperative MRI was used to assess tumor characteristics. Postoperative imaging, conducted within 3 months after surgery, along with the surgery report, was reviewed to confirm the extent of resection and edema resolution.

### 2.3. Tumor Resection Classification

The extent of resection (EoR) was based on the Simpson Classification [8]. The preoperative and postoperative tumor volumes were measured using the Smartbrush tool of semi-automatic segmentation software (BrainLAB^®^, Munich, Germany).

### 2.4. Questionnaire

As part of the subjective QoL assessment, the QOLIE-31 questionnaire, previously validated in clinical studies on epilepsy, was administered to patients online via the Google Forms platform [17,18,19]. At the time of survey completion, none of the patients were actively taking anti-epileptic drugs (AEDs). Patients were selected from the institutional meningioma database according to the aforementioned inclusion criteria. Each patient was initially contacted by telephone, during which the study was introduced, and participation was recommended. Patients were informed that their responses would be anonymized, and that participation was entirely voluntary. After obtaining verbal consent, the QOLIE-31 questionnaire was translated into German and then sent to patients via mail. The scale’s language equivalence was ensured. The questionnaire was also translated into other languages in previous studies, where it continued to show validated results [19,25,26].

### 2.5. Statistics

The raw data from patient responses were analyzed in accordance with the QOLIE-31 scoring system [27]. The QOLIE-31 questionnaire comprises seven subscales, each containing a set of items designed to assess specific aspects of QoL in meningioma patients with seizures. As part of the analysis, the untransformed, numerical raw values were transformed into a 0–100 scale. The scores for the individual subcategories of the questionnaire, as well as the total score, were dichotomized using the median and calculated on a scale of 0 to 100 points [28]. Before statistical analysis, all patient responses were validated and cross-checked for inconsistencies to ensure data accuracy. Furthermore, one point was awarded for the aspect of general health, which was not integrated into the subscales but contributed to the total score. The total score was defined as the mean of subscales. Subsequently, the total score was dichotomized for further statistical analyses. In this scoring system scale, a score of 100 indicated the highest possible level of functioning, while 0 represented the most unfavorable outcome. Depending on the distribution of the data, either a t-test or Mann–Whitney U-test was used. Receiver Operating Characteristics (ROC) curves were generated to assess the diagnostic accuracy of metric variables such as age, MIB-1 Index, and Karnofsky Performance Status (KPS) to identify patients with high or low QOLIE-31 scores. The Area Under the Curve (AUC) was calculated, and a 95% Confidence Interval (CI) was determined to evaluate the statistical reliability of these variables. Dexamethasone dosage and duration were standardized per institutional protocols (preoperative: 4–8 mg/day for ≤7 days; postoperative: tapered over 1–2 weeks). After performing the univariate analysis, a multivariate logistic regression was subsequently conducted. A *p*-value of <0.05 was considered statistically significant in the multivariate analysis. For the univariate analysis, a less stringent threshold of *p* < 0.10 was used to identify potential predictors for inclusion in the multivariate model. This approach allows for the detection of variables that may not show significance in univariate testing but could contribute to outcomes when adjusting for confounding factors. The Odds Ratios (OR) were reported along with their 95% CI, and a *p*-value below 0.05 was considered statistically significant in the multivariable analysis. All statistical analyses were performed using SPSS version 29.0 (IBM, Armonk, NY, USA). ROC curves were generated using SPSS. Forest plots and bubble plots were created using the R package ggplot2 (R Foundation for Statistical Computing, Vienna, Austria).

## 3. Results

### 3.1. Patient Selection

**Figure 1:**
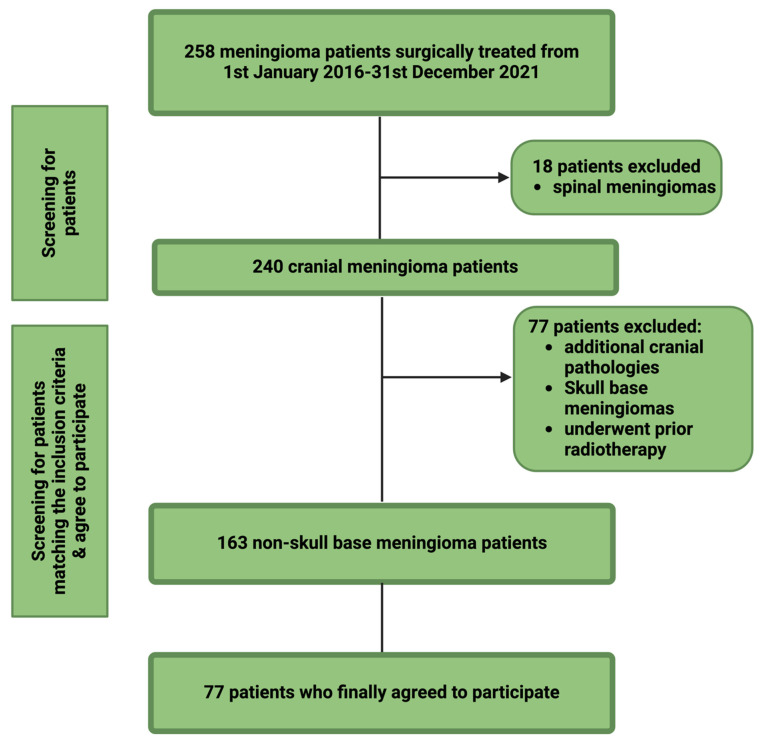
A total of 258 meningioma patients who underwent surgical treatment between 2016 and 2021 were screened. Of these, 18 patients were excluded due to spinal meningiomas, leaving 240 cases of cranial meningiomas. An additional 77 patients were excluded for reasons including the presence of additional intracranial pathologies, skull base meningiomas, or prior radiotherapy. Ultimately, 77 patients with non-skull base meningiomas and documented perioperative seizures were included in the final analysis.

Of the 258 meningioma patients surgically treated between January 2016 and December 2021, 18 were excluded for having spinal meningiomas, leaving 240 cranial meningioma cases. After further excluding 77 patients with additional cranial pathologies, skull base meningiomas, or prior radiotherapy, 77 of the remaining 163 non-skull base meningioma patients agreed to participate in the QOLIE-31 survey. Figure 1 summarizes the screening workflow.

### 3.2. Patient Characteristics

A total of 77 patients who experienced perioperative seizures with falx, parasagittal, and convexity meningiomas participated in this study. The median time from surgery to survey completion was 36 months (IQR: 12–60 months). The mean age at surgery was 61.8 years (SD ± 12.3). Among these patients, 33 (42.9%) were male and 44 (57.1%) were female. In terms of tumor location, 32 meningiomas (41.6%) were located along the falx, and 45 (58.4%) were located on the convexity. According to the WHO classification, 61 patients (79.2%) had grade 1, 14 (18.2%) had grade 2, and two patients (2.6%) had grade 3 meningiomas. Based on the Simpson resection classification, 38 patients (49.4%) underwent a Grade I resection, 33 (42.9%) had a Grade II resection, two (2.6%) had a Grade III resection, and four patients (5.2%) underwent a Grade IV resection.

Peritumoral edema was observed in 47 patients (61%), while 30 patients (39%) did not exhibit peritumoral edema. Regarding the neuroanatomical location, 36 meningiomas (46.8%) were in the right hemisphere, 32 (41.6%) were in the left hemisphere, and nine (11.7%) extended across both hemispheres. The mean time from diagnosis to surgery was 9.5 months (SD ± 16.6). The average length of stay in the intensive care unit (ICU) was 2.5 days (SD ± 4.7). A preoperative midline shift was observed in 13 patients, with an average shift of 0.7 mm (SD ± 0.5). In the perioperative management, 26 patients (33.8%) received dexamethasone preoperatively, whereas 51 patients (66.2%) did not. Postoperatively, dexamethasone was administered to 30 patients (39.0%), whereas 47 patients (61.0%) were not treated with it. Additionally, 61 patients (79.2%) presented with a dural attachment, with an average attachment length of 4.3 mm (SD ± 2.2). The mean hospital stay was 11.9 days (SD ± 7.9), and the median KPS was 70 (interquartile (IQR) range 70–80). Detailed patient and neuroradiological characteristics are provided in Table 1.

### 3.3. Univariate Analysis

The variables included in the univariate analysis, such as tumor location, peritumoral edema, patient age at surgery, preoperative KPS, WHO grade, Simpson grading scale, and postoperative complications, were selected based on their established relevance in predicting postoperative outcomes in meningioma patients. Among these, peritumoral edema, tumor location, and preoperative KPS have been widely recognized as strong predictors of seizure occurrence and postoperative functional outcome [3]. Specifically, peritumoral edema has been shown to increase seizure risk due to its irritative effect on the adjacent cortex. Tumor location, particularly in falx and convexity meningiomas, has been linked to higher seizure frequencies and postoperative neurological impairment. Preoperative KPS is frequently used as a marker of baseline functional status and has been associated with postoperative recovery and QoL outcomes [7]. Due to the absence of standardized and established cut-offs to assess improvement or deterioration in the QOLIE-31 score, we dichotomized the median total score. A value >49.78 was defined as a good postoperative QoL, while a value <49.78 was considered a poor postoperative QoL. Regarding the impact of seizures on QoL, 39 patients (50.6%) reported an improvement, while 38 patients (49.4%) reported a deterioration in QoL. Univariate analysis revealed a statistically significant association between age at surgery, MIB-1 index, preoperative KPS, and postoperative changes in QoL. Specifically, higher age at surgery was significantly associated with a lower total QoL score, indicating a negative impact on postoperative QoL (*p* = 0.034). A higher MIB-1 index also correlated significantly with a lower postoperative QoL score (*p* = 0.084). Additionally, the presence of peritumoral edema (*p* = 0.024) and tumor neuroanatomical localization (*p* = 0.016) showed significant associations with QoL outcomes. For tumor localization, a Chi-Square Test indicated that meningiomas located along the falx were associated with lower total QoL scores compared to convexity meningiomas (*p* = 0.016). A significant correlation was also found between lower preoperative KPS and poorer total QoL scores (*p* = 0.047).

No statistically significant associations were observed for time from diagnosis to surgery (*p* = 0.920), length of ICU stay (*p* = 0.671), tumor dural attachment length (*p* = 0.687), midline shift (*p* = 0.089), tumor sphericity (*p* = 0.134), length of hospitalization (*p* = 0.455), tumor volume (*p* = 0.480), preoperative dexamethasone administration (*p* = 0.579), postoperative dexamethasone administration (*p* = 0.311), tumor surface area (*p* = 0.697), WHO grade (*p* = 0.344), Simpson resection grade (*p* = 0.298) and postoperative complication rate (*p* = 0.577). Table 2 presents a summary of the univariate analysis comparing low and high QOLIE-31 scores.

**Figure 2:**
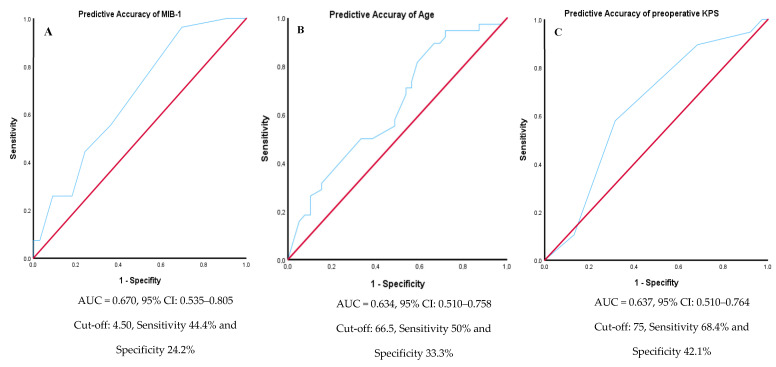
ROC curves illustrating the predictive accuracy of three variables for outcome prediction. (**A**) The ROC curve for MIB-1 index, showing an AUC of 0.670 with a 95% CI of 0.535 to 0.805. The optimal cut-off value was 4.50, yielding a sensitivity of 44.4% and a specificity of 24.2%. (**B**) The ROC curve for age at diagnosis, with an AUC of 0.634 with a 95% CI of 0.510 to 0.758. A cut-off of 66.5 years provided a sensitivity of 50% and a specificity of 33.3%. (**C**) The ROC curve for preoperative KPS, demonstrating an AUC of 0.637 with a 95% CI of 0.510 to 0.764. A cut-off of 75 achieved a sensitivity of 68.4% and a specificity of 42.1%.

ROC curve analysis was used to assess the predictive accuracy of age at surgery, MIB-1 index, and preoperative KPS. The AUC for age at surgery was 0.634 (95% CI: 0.510–0.758), with a sensitivity of 50% and specificity of 33.3%, identifying an optimal age cut-off of 66.5 years. The MIB-1 index yielded an AUC of 0.670 (95% CI: 0.535–0.805), with a sensitivity of 44.4% and specificity of 24.2%, at a cut-off of 4.50. The preoperative KPS score demonstrated an AUC of 0.637 (95% CI: 0.510–0.764), with a sensitivity of 68.4% and specificity of 42.1%, and an optimal cut-off KPS score of 75. ROC curves are shown in Figure 2.

### 3.4. Multivariate Analysis

Following the univariate analysis, variables with *p* < 0.10 were included in a multivariate logistic regression model to determine independent predictors of postoperative QoL. Significant correlations were found between several variables and postoperative quality of life (QoL), including age at surgery, MIB-1 index, preoperative KPS, presence of peritumoral edema, and meningioma location along the falx. These variables were subsequently included in a multivariate regression analysis to evaluate their independent effects on postoperative QoL. For this analysis, age at surgery, MIB-1 index, and preoperative KPS were dichotomized. Due to a *p*-value of 0.024 in the univariate analysis, peritumoral edema was included in the multivariate analysis due to its well-documented association in the literature with seizure occurrence and neurological outcomes in meningioma patients [7]. After adjusting for other covariates, the multivariate analysis indicated that the presence of peritumoral edema was an independent factor of reduced postoperative QoL (OR = 3.89, 95% CI: 1.04–14.47., *p* = 0.043). Patients with peritumoral edema had nearly four times higher odds of experiencing reduced postoperative QoL.

**Figure 3:**
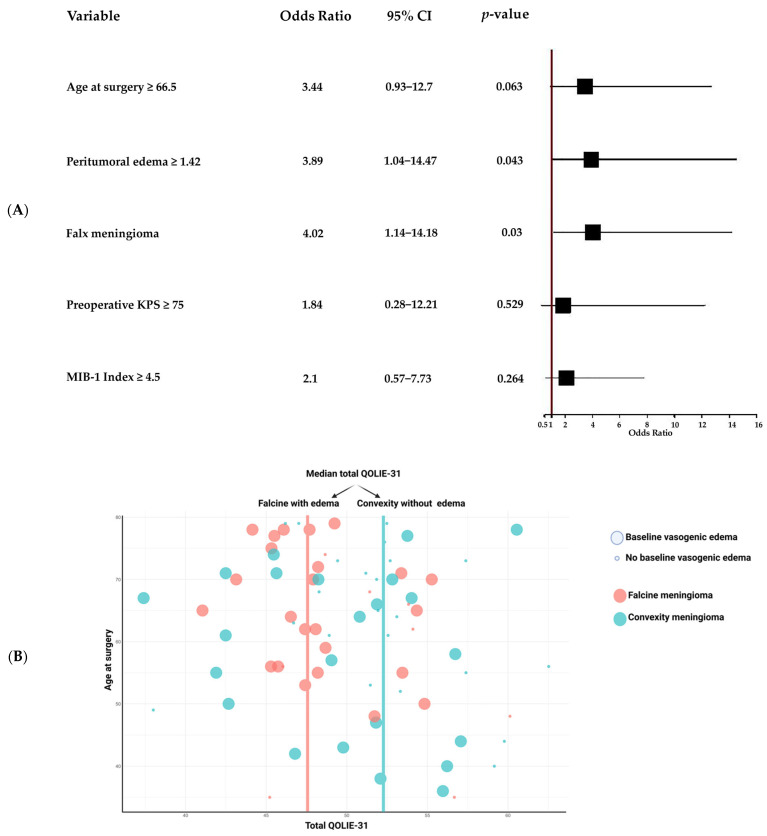
(**A**) Forest plot of factors associated with outcomes after meningioma surgery. The forest plot displays odds ratios (ORs) with 95% confidence intervals (CIs) from a multivariable logistic regression analysis examining factors associated with surgical outcomes. The red dashed line at OR = 1.00 represents the null value (no effect). Significant predictors include peritumoral edema (cut-off ≥1.42) with OR = 3.89 (95% CI: 1.04 – 14.47, *p* = 0.043) and falx meningiomas, which show OR = 4.02 (95% CI: 1.14 – 14.18, *p* = 0.030). Non-significant factors include age at surgery (cut-off ≥66.5) (OR = 3.44, 95% CI: 0.93 – 12.70, *p* = 0.063), preoperative KPS (cut-off ≥75) (OR = 1.84, 95% CI: 0.28 –12.21, *p* = 0.529), and MIB-1 Index (cut-off ≥4.5) (OR = 2.10, 95% CI: 0.57 – 7.73, *p* = 0.264). Significant factors (peritumoral edema and falx meningiomas) demonstrate strong predictive relevance, while non-significant factors show CIs crossing the reference line (OR = 1). (**B**) Bubble plot comparing total QOLIE-31 scores and age at surgery. This bubble plot examines the relationship between total QOLIE-31 score (*x*-axis) and age at surgery (*y*-axis). Tumor locations are color-coded: red for falx meningiomas and turquoise for convexity meningiomas. Bubble size indicates the presence of baseline vasogenic edema, with larger bubbles representing its presence and smaller bubbles its absence. Blue-bordered bubbles indicate patients with edema, and black-bordered bubbles represent those without. The red line in the bubble plot represents the median total QOLIE-31 score for falcine meningiomas with edema, with a median value of 47.80. The turquoise line represents the median total QOLIE-31 score for convexity meningiomas, with a median value of 52.14. The plot highlights variability in QoL scores across tumor types, age groups, and edema status, suggesting complex interdependencies influencing postoperative QoL outcomes.

Falx meningiomas were also a statistically significant independent predictor of lower postoperative QoL (OR = 4.02, 95% CI: 1.14–14.18, *p* = 0.03). Patients with falx meningiomas were also approximately four times more likely to experience worse postoperative QoL compared to those with convexity meningiomas. However, the MIB-1 proliferation index, preoperative KPS, and higher age at surgery did not reach statistical significance in the multivariate analysis. Figure 3A,B provide a visual summary of the multivariate results.

## 4. Discussion

The aim of the present study was to investigate the QoL of patients with falx and convexity meningiomas who underwent surgical therapy. This study exclusively enrolled patients with perioperative seizures to evaluate the impact of seizures on postoperative QoL. By restricting inclusion to this population, we isolated the relationship between seizure burden and postoperative QoL. For this purpose, we used the QOLIE-31 questionnaire, which has demonstrated reliability and validity in multiple clinical studies [17,18,19]. By ensuring that no patients were on AEDs during the QoL assessment, we eliminated confounding effects of medication side effects (e.g., cognitive fatigue, mood changes) on reported outcomes. However, it has not yet been evaluated for meningioma patients so far. This strengthens the validity of our findings, as QoL impairments can be more confidently attributed to residual edema or tumor location rather than pharmacological factors.

Previous research has largely employed general HRQoL tools, such as the SF-36, EORTC QLQ-BN20, and EORTC QLQ-C30, to assess QoL in meningioma patients [29,30,31]. Although these tools provide valuable insights into overall well-being, they lack the specificity required to capture epilepsy-related QoL outcomes. Englot et al. demonstrated in their meta-analysis that both preoperative and postoperative tumor edema are robust predictors of epileptic seizures, with a notable negative impact on QoL [7]. While both falx and convexity meningiomas can cause edema, falx meningiomas often lead to more substantial edema, which increases the risk of both preoperative and postoperative seizures. Although edema is also present in convexity meningiomas, the ability to achieve Gross Total Resection (GTR) in these cases often helps to mitigate seizure risk more effectively [10,13,14]. Despite the potential confounding effects of dexamethasone on edema and QoL, adjusted analyses confirmed that edema itself, and not steroid use, was the primary driver of reduced QoL. Our findings align with these observations, as peritumoral edema also emerged as a significant predictor of postoperative seizures and reduced QoL in our study. Prior studies have highlighted that even minor edema surrounding non-skull base meningiomas can lead to cortical irritation and increased seizure susceptibility. Additionally, the distribution of edema, particularly in eloquent or seizure-prone cortical regions, may be more critical than the absolute volume alone in determining neurological impact [3]. Additionally, peritumoral edema emerged as an independent predictor of reduced QoL, even after adjusting for confounders in the multivariate model. These and our findings align with previous studies demonstrating the deleterious effects of brain edema on seizure burden and functional outcomes [7].

The neurosurgical management of meningiomas, especially in differentiating between falx and convexity tumors, presents unique challenges. Falx meningiomas, due to their proximity to the falx cerebri and critical structures like the superior sagittal sinus, are often more challenging to resect fully. This anatomical complexity leads to a higher rate of subtotal resections (STR), which is associated with an increased likelihood of postoperative complications and poorer seizure control, potentially impacting postoperative QoL [3,22]. In contrast, convexity meningiomas are generally more accessible, allowing for a higher likelihood of GTR, which is associated with improved seizure control and better QoL outcomes [14,32]. While no studies have directly compared Simpson resection grade with postoperative QoL, and although Simpson grade I/II resection may reduce recurrence rates and subsequent surgeries [33], our study found no significant association between Simpson classification and postoperative QoL. Based on our findings and the literature, this may suggest that QoL is influenced more by factors such as perioperative complications (e.g., seizures) and preoperative functional status [34].

The present study demonstrated that higher age at surgery was associated with lower QOLIE-31 scores and reduced QoL in meningioma patients. This finding aligns with the current literature. In a study of 133 meningioma patients, Timmer et al. showed that higher age at surgery was associated with poorer postoperative HRQoL [35]. Another study by Li et al., involving 470 patients, also highlighted that increasing age was an independent predictor for reduced QoL and functional outcomes in meningioma patients [36].

The findings of this study emphasize the need for tailored, patient-specific therapeutic strategies for perioperative and long-term postoperative management to improve QoL in meningioma patients, particularly those with falx and convexity tumors prone to seizures. The association of higher age at surgery, peritumoral edema, and tumor location along the falx with poorer postoperative QoL highlights the importance of comprehensive preoperative assessments. Furthermore, as higher age and edema significantly impact QoL, future studies should explore targeted interventions, such as edema reduction strategies and personalized rehabilitation plans for older patients.

There are also some limitations in the present study. First, this study was conducted in a single neurosurgical institution, which may limit the generalizability of the results based on different experiences in other neurosurgical centers. Second, the sample size was relatively small, which may affect the power of this study to detect smaller differences in QoL outcomes. While our findings highlight key factors influencing postoperative QoL, larger prospective multicenter studies are needed to confirm these results. Additionally, our study included only patients who experienced perioperative seizures, which may limit broader applicability to all meningioma patients. Nevertheless, our findings emphasize the need for targeted edema-reduction strategies beyond conventional steroid regimens. Additionally, because this study relies on subjective patient-reported outcomes, there is a potential for response bias, as patients’ perceptions and willingness to report their quality of life may vary based on individual experiences, expectations, or psychological factors. Another limitation of our study is the exclusion of brain invasion from our statistical analysis. While brain invasion has been identified as a risk factor for seizures in meningioma patients, only two patients in our cohort exhibited this characteristic. Due to the extremely small sample size, statistical analysis would not have yielded reliable conclusions. Therefore, while this is an important factor, its impact could not be assessed in our study. A key methodological limitation of our study is the lack of a control group with seizure-free patients. As a result, we are unable to make direct comparisons between patients with and without perioperative seizures, which limits the generalizability of our findings. Future studies should incorporate prospective comparison groups with and without seizures to provide a more comprehensive understanding of the factors affecting postoperative QoL in meningioma patients.

## 5. Conclusions

In conclusion, the present study highlights that peritumoral edema and tumor location along the falx are significant independent predictors of poorer postoperative QoL in patients with perioperative seizures. These findings underscore the need for targeted and individual strategies to address these factors, particularly in optimizing neurosurgical approaches and postoperative patient care. Future research is urgently needed and should include larger, multi-center studies with extended follow-up to better understand the long-term impact of these factors on patients’ QoL outcomes. Specifically, further exploration of targeted interventions for edema reduction and neuroprotective strategies for meningioma patients is warranted to improve patient-centered care.

## Figures and Tables

**Table 1:** Patient characteristics.

Characteristics	Value
Mean age at surgery	61.8 (SD ± 12.3)
<64	37/77 (48.1%)
≥64	40/77 (51.9%)
Sex	
Female	44/77 (57.1%)
Male	33/77 (42.9%)
Median MiB-1 index	3% (IQR 3–5)
WHO classification	
1	61/77 (79.2%)
2	14/77 (18.2%)
3	2/77 (2.6%)
Simpson grade	
I	38/77 (49.4%)
II	33/77 (42.9%)
III	2/77 (2.6%)
IV	4/77 (5.2%)
Peritumoral edema	
Present	47/77 (61.0%)
Absent	30/77 (39.0%)
KPS preoperative median (IQR)	70 (70–80)
Mean time from diagnosis to surgery in months	9.5 (SD ± 16.6)
Mean time of ICU length of stay in days	2.5 (SD ± 4.7)
Neuroanatomical location	
Falx/parasagittal	32/77 (41.6%)
Convexity	45/77 (58.4%)
Dural attachment	
Present	61/77 (79.2%) average attachment length of 4.3 cm (SD ± 2.2)
Absent	16/77 (20.8%)
Mean length of hospitalization in days	11.9 (SD ± 7.9)
Sphericity median (IQR)	0.92 (0.87–0.95)
Tumor volume median (IQR) in cm^3^	22.0 (9.5–38)
Tumor surface area (IQR) in mm^2^	4447.7 (25–6.9)
Preoperative dexamethasone administration	
Yes	26/77 (33.8%)
No	51/77 (66.2%)
Postoperative dexamethasone administration	
Yes	30/77 (39.0%)
No	47/77 (61.0%)
Postoperative complications	
Yes	30/77 (39.0%)
No	47/77 (61.0%)
Brain invasion	
Yes	2/77 (2.6%)
No	75/77 (97.4%)

**Table 2:** Univariate analysis of patient- and disease-specific characteristics among those with low or high QOLIE-31.

Characteristics	Low QOLIE-31 (<49.78, n = 38)	High QOLIE-31 (>49.78, n = 38)	*p*-Value
Age at surgery (mean ± SD)	64.8 (± 10.9)	58.9 (± 12.9)	0.034
Location			0.016
Falcine	21/77	11/77	
Convexity	17/77	28/77	
MIB-1 index (mean ± SD)	6.6 (± 6.6)	4.1 (± 3.1)	0.084
WHO grade			0.344
1	29/77	32/77	
2	7/77	7/77	
3	2/77	0/77	
Simpson grading scale			0.298
1	17/77	21/77	
2	18/77	15/77	
3	0/77	2/77	
4	3/77	1/77	
Peritumoral edema			0.024
Present	28/77	19/77	
Absent	10/77	20/77	
KPS preoperative (mean ± SD)	70.3 (± 11.9)	75.3 (± 9.5)	0.047
Time from diagnosis to surgery in months (mean ± SD)	9.3 (± 17.9)	9.6 (± 15.5)	0.920
ICU length of stay in days (mean ± SD)	2.2 (± 4.2)	2.7 (± 5.1)	0.671
Dural attachment in cm (mean ± SD)	4.4 (± 2.5)	4.2 (± 1.9)	0.687
Midline shift in cm (mean ± SD)	1 (± 0.6)	0.5 (± 0.3)	0.08
Sphericity (mean ± SD)	0.8 (± 0.2)	0.9 (± 0.1)	0.134
Tumor surface area in mm^2^ (mean ± SD)	5.460 (± 3.390)	6.002 (± 6.527)	0.697
Tumor volume in cm^3^ (mean ± SD)	28.3 (± 22.5)	34.3 (± 39.0)	0.480
Postoperative complications			0.577
Yes	16/77	14/77	
No	22/77	25/77	
Preoperative dexamethasone administration			0.579
Yes	14/77	12/77	
No	24/77	27/77	
Postoperative dexamethasone administration			0.311
Yes	17/77	13/77	
No	21/77	26/77	

## Data Availability

The datasets generated and analyzed during the current study are available from the corresponding author upon reasonable request. Due to ethical and privacy considerations, the data cannot be made publicly accessible but will be shared upon request in accordance with institutional and regulatory guidelines. Anonymized patient data and relevant statistical analysis files will be provided to qualified researchers for non-commercial academic purposes.
